# Drug Utilization Evaluation of Imipenem and Intravenous Ciprofloxacin in a Teaching Hospital

**Published:** 2013

**Authors:** Sarah Mousavi, Mehdi Behi, Mohammad Reza Taghavi, Alireza Ahmadvand, Shadi Ziaie, Mandana Moradi

**Affiliations:** a *Clinical Pharmacy Department, School of Pharmacy, Tehran University of Medical Sciences , Tehran, Iran.*; b *School of Pharmacy, Zabol University of Medical Sciences, Zabol, Iran. *; c *Faculty of Medicine, Zabol University of Medical Sciences, Zabol, Iran.*; d *Consultant and researcher, Research Center for Rational Use of Drugs, Tehran, Iran.*; e *Clinical Pharmacy Department, School of Pharmacy, Shahid Beheshti University of Medical Sciences, Tehran, Iran. *

**Keywords:** Drug Utilization Evaluation, Imipenem, Ciprofloxacin, Hospital

## Abstract

Drug Utilization Evaluation (DUE) studies are designed to assess drug usage appropriateness**. **We aim to evaluate the drug utilization of intravenous ciprofloxacin and imipenem, two of the broad spectrum antibiotics that consume a significant proportion of our hospitals’ outlay, in different wards of a teaching hospital in Zabol. During a 5 months period (December 2010 to May 2011), 263 patients who received imipenem or intravenous ciprofloxacin were assigned to this study. Retrospective review of patient’s records was carried out. Data were converted to Defined Daily Dose (DDD) and the ratio of prescribed daily dose per DDD was calculated. Among these records, 100 patients received either imipenem or ciprofloxacin. The ratio of prescribed daily dose to DDD was 1.5 for both antibiotics. Almost all patients received empiric therapy in both groups. Only 13 patients (26%) in ciprofloxacin group and 4 patients (8%) in imipenem group received their antibiotics consistent with American Hospital Formulary System (AHFS) mentioned indication. Baseline Blood Urea Nitrogen (BUN) and serum Creatinine were ordered for only 37 patients (74%) in both groups with 15 abnormal results but dose adjustment performed just in one case with decreased renal function. In conclusion**, **the majority of courses with both drugs were empirically selected and continued and required lab tests for drug monitoring and dose adjustments were not performed in most cases. Educational interventions, developing a local formulary and a strict antibiotic prescribing policy for example by prior approval by an infectious disease consultant can help significantly to overcome these problems.

## Introduction

Drug utilization evaluation (DUE) as an effective tool for monitoring the appropriateness of the usage of various medications (1) is an essential component of pharmacy service provision, and clinical pharmacy practice (2- 4). DUE is a structured process to analyze the pattern of drug administration in various practice settings, including hospitals in relation to guidelines or predetermined standards. DUE programs will maintain the interventions that will improve patient outcomes (5).

Antibiotics are among the most widely used class of drugs in hospitals (6) and they are really important to be used optimally (7) otherwise emerging resistant pathogens will interfere with treatment outcome (8). From this perspective, it is crucial to carry on DUE about antibiotics. It can help to identify actual and potential drug-related problems, prevent the development of drug resistant organisms and control treatment costs (9).

This study was conducted in order to evaluate and improve appropriate use of imipenem and intravenous ciprofloxacin, two of the broadest-spectrum antibiotics that consume a significant proportion of most hospitals’ outlay on antimicrobial agents. Based on our initial evaluation 1455 patients received imipenem (n = 655) or intravenous ciprofloxacin (n = 800) from March to May 2010 in our hospital, considering high usage of them. There are small number of DUE’s retrieved these target drugs and as far as we know there is not any DUE performed in Zabol city, Sistan- Baluchestan province.

## Experimental

*Methods*

*Setting*

The study was a cross-sectional DUE study, carried out in different wards in Amir Hospital, affiliated to Zabol University of Medical Sciences (ZUMS). This hospital is the only hospital providing care in Zabol city and its regions. Two hundred and sixty-three hospital beds were assigned to this study.

*Study subjects*

All patients, for whom intravenous ciprofloxacin and imipenem or combinations of them were prescribed at Amir Hospital, during a 5 months period, from December 2010 to May 2011, were included in this study. From the hospital information system, the record numbers of all patients that received imipenem or intravenous ciprofloxacin during the specified period were obtained. The patient’s charts were retrieved and a retrospective review of these records was carried out.

*Data collection*

Relevant information from each patient’s chart was obtained. The data were recorded in a predesigned data collection form. All data extraction was carried out by a pharmacist, and whenever data extraction and interpretation was unclear, a clinical pharmacist was consulted to arrive at a consensus.

*Study indicators*

Demographic and clinical data were retrieved from the relevant patient’s chart.

The admission variables included name of ward, length of hospital stay, history of drug allergy, first and final diagnoses. The drug indicators used included indication, dosing regimen, microbiological culture/sensitivity testing and combination therapy regimen.

Outcome indicators included the clinical outcome and the occurrence of adverse drug reaction.

*Audit criteria*

The appropriateness of imipenem and ciprofloxacin usage was assessed according to the culture results and based on the indication that was mentioned in American Hospital Formulary Systems (AHFS) book (10).

Data were converted to Defined Daily Dose (DDD), according to Anatomic and therapeutic chemical classification system (ATC/DDD). Defined Daily Dose is a unit based on the average daily dose used for main indication for consumption of certain medication (11). Ratio of prescribed daily dose per DDD was calculated.

Descriptive analyses of data were performed using SPSS software (version, 16).

## Results

During the study period, 27559 cases have been reviewed from hospital information system. Among these records, 100 patients received either imipenem or ciprofloxacin (50 in each group). The demographic of patients and distribution of antibiotics among different ward are presented in Table 1. Of the ciprofloxacin used, 29 courses (58%) were administered in internal medicine wards. Imipenem was mostly used in gastroenterology ward (46%).

**Table 1:** Characteristics of patients and distribution of antibiotics in different wards

**Demographic**	**Intravenous Ciprofoloxacin N(%)**	**Imipenem N(%)**
Sex (M/F)	21/29	23/27
Age*	54.5 ± 16.81	55.58 ± 23.5
Wards		
Pediatrics	0 (0)	2 (4)
Internal	29 (58)	13 (26)
Infectious disease	1 (2)	2 (4)
Gastrointestinal	8 (16)	23 (46)
Men Surgical ward	4 (8)	1 (2)
Women Surgical ward	3 (6)	3 (6)
Emergency	1 (2)	2 (4)
Cardiac Care Unit	2 (4)	0 (0)
Others	2 (4)	4 (8)

* Mean ± SD

The median duration of treatment with ciprofloxacin and imipenem was 4 days (range 1-11 and 1-22 days respectively).

 The length of hospital stay (mean ± SD) was 8.46 ± 3.92 days in ciprofloxacin group and 8.08 ± 4.67 days in imipenem group.

The mean dosage regimen in ciprofloxacin group was 745 mg/day in adults (> 12 years old). Imipenem was administered 1306mg/day for children (< 12 years old) and 1540 mg/day in adults. The ratio of prescribed daily dose to DDD was 1.5 for both antibiotics. Table 2 shows appropriate antibiotic therapies in terms of dosing, interval and duration of treatment.

**Table 2:** Appropriateness of imipenem and ciprofloxacin therapy in 50 patients in each group.

**Appropriate utilization**	**Ciprofloxacin N (%)**	**Imipenem N (%)**
Maintenance dose	43(86%)	48(96%)
Dosing interval	50(100%)	41(82%)
Duration of treatment	5 (10%)	4(8%)

Among the 50 courses, ciprofloxacin was prescribed for empiric therapy in 50 cases (100%). One antibiogram was performed in this group without ciprofloxacin disk.

**Figure 1:**
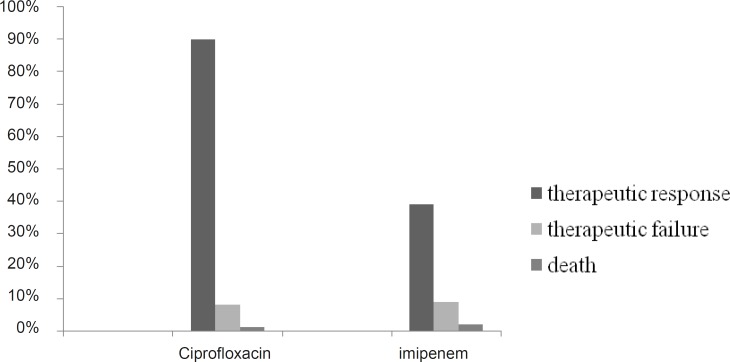
Clinical outcomes of patients

About 98% of patients received imipenem empirically and targeted therapy in 1 case (2%). Figure 1 show the clinical outcomes of patients.

About 90% of our patients responded to ciprofloxacin while only 39% responded to imipenem. In 30% of patients who treated with ciprofloxacin final diagnoses was different from admission diagnoses compare to 32% in imipenem group. Table 3 shows how admission diagnoses changed. Most frequent admission diagnosis was pneumonia in imipenem (21 cases) and diabetic foot (10 cases) in ciprofloxacin group. Large number of final diagnoses could not be categorized as infectious diseases (34% in ciprofloxacin and 28% in imipenem group).

**Table 3:** Changes in admission diagnosis

**Imipenem**		**Ciprofloxacin**	
**Primary diagnoses**	**Final diagnoses**	**Primary diagnoses**	**Final diagnoses**
Pneumonia	Foreign object	Pneumonia	Tuberculosis
Chronic Obstructive Pulmonary Disease(COPD)	Pneumonia	Chronic Obstructive Pulmonary Disease(COPD) (2)	Pneumonia(2)
Abdominal pain	Abdominal wall hematoma	Abdominal pain	Cholangitis
Abdominal pain	Chronic Fistula	Abdominal pain	Abdominal wall hematoma
Abdominal pain	Cholangiocarcinoma	Vomiting	Chronic Kidney Disease
Respiratory distress	Pulmonary edema	Gastroenteritis	Celiac disease
Dyspnea	Encephalopathy	Urinary Tract Infection	Pre Menstrual Syndrome
Dyspnea(3)	Ascites(3)	Flank pain	Cholecystitis
Dyspnea	Tuberculosis	Pneumonia	Chronic Heart Failure
Cirrhosis	Ischemic Heart Disease	Acute abdomen	Ascites
Loss of Consciousness	Cerebro Vascular Accident	Loss of Consciousness	Acute Renal Failure
Fever	Peptic Ulcer Disease	Gastrointestinal bleeding	Gastric Adenocarcinoma
Constipation	Bowel obstruction	Acute abdomen	Acute appendicitis

During the study period, only 17 patients (34%) in ciprofloxacin group and 7 patients (14%) in imipenem group had bacterial culture results. Among them, 7 cultures in ciprofloxacin group and 3 cultures in imipenem group were positive. Sensitivity test (antibiogram) was performed only for 1 patient in each group.

Baseline Blood Urea Nitrogen (BUN) and serum Creatinine (Cr) was ordered for 37 patients (74%) in both groups. The results reported abnormal (ClCr < 75 mL/min) in 9 patients of imipenem and 6 of ciprofloxacin group. Further renal function tests performed only in 3 patients who were on imipenem and 6 patients on ciprofloxacin who had abnormal baseline BUN and Cr. Dose adjustment performed just in one case in imipenem group from all 15 patients (in both group) with decreased renal function.

Table 4 shows the antibiotics which were prescribed before, concurrently and after the ciprofloxacin and imipenem. Antibiotics used most commonly with ciprofloxacin were ceftriaxon and gentamicin. One case of double beta-lactam therapy was used in ciprofloxacin group. In imipenem group ceftriaxone was the most administered drug in combination.

**Table 4:** Antibiotics administered before, concurrent and after ciprofloxacin and imipenem

**Ciprofloxacin**			**Imipenem**		
**Before**	**Concurrent**	**After**	**Before**	**Concurrent**	**After**
Ceftriaxon +Clindamycin	Azithromycin+Metronidazole	Ceftriaxon	Ceftriaxon	Anti tuberculosis	Azithromycin
Ceftriaxon +Azithromycin	Cefotaxim+ Metronidazole	Ceftriaxon+Cloxacillin	Ceftriaxon +Clindamycin	Azithromycin+ Metronidazole	Cefepim
Cefotaxim+Metronidazole	Ceftriaxon	Metronidazole	Ceftriaxon + Clindamycin+ Antituberculosis	Azithromycin+Ceftriaxon+Metronidazole	Ceftriaxon +Azithromycin
Ceftriaxon+ Clindamycin+Metronidazole	Ceftriaxon + Metronidazole		Ceftriaxon+Methronidazl+ Co-trimoxazole	Cefazolin	Metronidazole
Ceftriaxon+ Cefotaxim+Metronidazole	Ceftriaxon + Clindamycin		Ceftriaxon+Metronidazole	Ceftriaxon + Metronidazole	
Ceftriaxon	Ceftriaxon+Azithromycin+Clindamycin		Clindamycin	Ceftriaxon + Clindamycin	
Metronidazole	Ceftriaxon+ Clindamycin+Metronidazole			Ceftriaxon+ Gentamycin	
Ceftriaxon +Metronidazole	Ceftriaxon+ Cefotaxim+Metronidazole			Ceftriaxon+ Methronidaz+Co-trimoxazole	
Clindamycin	Clindamycin			Ceftriaxon+Metronidazole+Cefazolin	
Clindamycin+ Azithromycin	Metronidazol+ Cefazolin				
Clindamycin + Penicillin	Metronidazole				
Clindamycin + Gentamycin					
Clindamycin+ Metronidazole					
Gentamycin					

Only 13 patients (26%) in ciprofloxacin group and 4 patients (8%) in imipenem group received their antibiotics consistent with AHFS mentioned indication. During the audit period, no adverse drug reactions were recorded in patient’s charts.

## Discussion

The results of this study show that imipenem and intravenous ciprofloxacin are mostly used empirically in our hospital without appropriate monitoring.

To minimize the emergence of resistant bacteria, antibiotic needs to be restricted to appropriate indications. Our results showed that most antibiotic courses in our hospital were empirically selected based on clinical judgment, and only minorities were based on relevant culture results. Some of previous DUEs about other broad-spectrum antibiotics had also shown that the vast majority of courses were empirically selected and continued (12). Vazin et al showed the length of empiric therapy with vancomycin was inappropriate in 50% of the patients (7). The majority of culture tests were ordered without antibiogram that make these culture results hard to interpret, performing of just one antibiogram emphasize this point.

The principal findings of our study were as follows. First, imipenem was generally administered in gastroentrology wards but the most diagnoses recorded in patient files were pneumonia that can reflect problems in patients triage in our hospital. Duration of treatment was 4 days in about 50% of our patients which seems logical, while during this time laboratory results may not be expected that often lead to adjustment of treatment.

In the current study, 58% of cases received ciprofloxacin while in the internal medicine wards. As the internal medicine ward patients may suffer from multiple disease, there may not be clear-cut indications for being prescribed ciprofloxacin and this drug usually used synergistically with other drugs, however about 90% of patients responded to ciprofloxacin but it is hard to say that this was the direct effect of ciprofloxacin.

Ciprofloxacin was prescribed every 12 h in all patients and imipenem every 6-8 h in most patients that are the recommended dose interval (13), calculated based on pharmacokinetic characteristics of these drugs. 

The ratio of prescribed daily dose to DDD was 1.5 for both antibiotics and it indicates that these antibiotics prescribed more than recommended daily dose, that can either reflect the prescribers concept about high rate of antimicrobial resistance and non-response to standard dosage or it can be simply the result of malpractice that need to be reviewed, as over treatment can increase the cost of treatment course and related adverse drug effects.

Regarding drug monitoring, we observed that although both antibiotics need dose adjustment in renal failure, baseline BUN and serum Cr assessment was not performed for all patients and even in patients with abnormal renal function drug doses were not adjusted. It reflects neglecting monitoring parameters in our practice setting.

As evidenced in a study performed by Takhar *et al. *(14), our results also showed, increased trend of polypharmacy in our hospital that has to be controlled by establishing empirical antibiotic guidelines. Antibiotics used most commonly with ciprofloxacin in our study subjects were ceftriaxone and gentamicin, as these three antibiotics have almost the same antimicrobial coverage; these combinations would not obtain optimal coverage for empiric therapy (15).

In about 30% of patients the first and final diagnosis was different, although most final diagnoses were not infectious which indicated that most patients didn’t need antibiotics and received it inappropriately.

No adverse drug reaction was recorded in patient files. Considering the rate and types of these reactions to object drugs (13), it could be the reason of lacking systematic reporting manual for adverse drug reaction in our hospital.

In conclusion, Educational interventions emphasizing rational antibiotic prescribing, along with effort to develop an updated local formulary, and a strict antibiotic prescribing policy for example by prior approval by an infectious disease consultant can help significantly to overcome these problems and to reduce the extent of resistance to antibiotics.

*Study limitation*

Some of our study limitations are as follows: The first concerns the guidelines for appropriate use of the Broad-spectrum antimicrobials in our hospital. Second, appropriateness was evaluated retrospectively based on patient’s files and some data may not be recorded. Third, neither seasonal nor physician variations in prescribing patterns were evaluated in this study, and the results obtained, represent the overall prescribing pattern in the hospital.
